# TGF-β autocrine pathway and MAPK signaling promote cell invasiveness and *in vivo* mammary adenocarcinoma tumor progression

**DOI:** 10.3892/or.2012.1813

**Published:** 2012-05-14

**Authors:** MARÍA CECILIA DAROQUI, PAULA VAZQUEZ, ELISA BAL DE KIER JOFFÉ, ANDREI V. BAKIN, LYDIA I. PURICELLI

**Affiliations:** 1 Department of Oncology, Montefiore Medical Center, Bronx, NY 10467; 2 Department of Cancer Genetics, Roswell Park Cancer Institute, Buffalo, NY 14263, USA; 3 Research Area, Institute of Oncology ‘Ángel H. Roffo’, 1417 Buenos Aires, Argentina

**Keywords:** transforming growth factor-β, migration, invasion, matrix metalloproteinase-9, actin cytoskeleton, mammary adenocarcinoma

## Abstract

Breast cancer progression and metastasis have been linked to abnormal signaling by transforming growth factor-β (TGF-β) cytokines. In early-stage breast cancers, TGF-β exhibits tumor suppressor activity by repressing cell proliferation and inducing cell death, whereas in advanced-stage tumors, TGF-β promotes invasion and metastatic dissemination. The molecular mechanisms underlying pro-oncogenic activities of TGF-β are not fully understood. The present study validates the role of TGF-β signaling in cancer progression and explores mediators of pro-oncogenic TGF-β activities using the LM3 mammary adenocarcinoma cell line, derived from a spontaneous murine mammary adenocarcinoma. Expression of kinase-inactive TGF-β receptors decreased both basal and TGF-β-induced invasion. Analysis of signal transduction mediators showed that p38MAPK and MEK contribute to TGF-β stimulation of cell motility and invasion. TGF-β disrupted the epithelial actin structures supporting cell-cell adhesions, and increased linear actin filaments. Moreover, MEK and p38MAPK pathways showed opposite effects on actin remodeling in response to TGF-β. Blockade of Raf-MEK signaling enhanced TGF-β induction of actin stress-fibers whereas p38MAPK inhibitors blocked this effect. A novel observation was made that TGF-β rapidly activates the actin nucleation Arp2/3 complex. In addition, TGF-β stimulated matrix metalloproteinase MMP-9 secretion via a MAPK-independent pathway. Experiments using syngeneic mice showed that kinase-inactive TGF-β receptors inhibit the first stages of LM3 tumor growth *in vivo*. Our studies demonstrate that autocrine TGF-β signaling contributes to the invasive behavior of mammary carcinoma cells. Moreover, we show that both MAPK-dependent and -independent pathways are necessary for TGF-β-induced effects. Therefore, MEK-ERK and p38 MAPK pathways are potential venues for therapeutic intervention in pro-oncogenic TGF-β signaling.

## Introduction

Dissemination of the primary tumor is the main cause of death in breast cancer patients. The dissemination process involves a series of distinct steps in which tumor cells migrate from the primary tumor, spread through lymphatic and blood vessels, and establish secondary tumors at distant sites (1). Accumulating evidence implicates transforming growth factor beta (TGF-β) cytokines in the control of tumor progression and dissemination. TGF-β cytokines repress tumor growth at early phases of tumorigenesis, in part by inhibiting cell-cycle progression and inducing cell death, but they are also able to promote tumor invasion and metastatic dissemination in late-stage tumors (2,3). Pro-tumorigenic TGF-β activity has been linked to the induction of epithelial-mesenchymal transition (EMT), cell motility and matrix-degrading enzymes. In addition, TGF-β may promote tumor progression by repressing the immune response (4), and by stimulating angiogenesis via upregulation of the pro-angiogenic factors VEGF and matrix metalloproteinase MMP-9 (5–7). Blockade of the soluble TGF-β ligand impairs tumor invasion and metastasis, further supporting the active role of TGF-β in cancer progression (8,9). However, TGF-β receptors and Smad transcription factors are frequently altered in cancer, and this has been associated with poor prognosis (10,11). The dual role of TGF-β in cancer complicates the development of therapies targeting TGF-β (12). Thus, unraveling the intracellular pathways and factors involved in TGF-β pro-oncogenic activities is critical for the development of putative anticancer TGF-β therapies.

TGF-β signal transduction is initiated by binding TGF-β cytokines to TGF-β type I and type II receptors (TβRI and TβRII), a complex of transmembrane glycoproteins with serine-threonine kinase activity (3). Upon ligand binding, TβRII phosphorylates TβRI, thus activating the TβRI kinase, which in turn phosphorylates and activates Smad transcription factors. Receptor-associated Smad2 and Smad3 (R-Smads) together with the co-mediator Smad4 translocate to the nucleus, where they regulate the transcription of TGF-β target genes. In addition, it has been shown that TGF-β can activate MAP kinases as well as PI3K-Akt signaling, contributing to the TGF-β effects on malignant tumor cells (3).

Cell adhesion, motility and invasion which are crucial for the metastatic process, depend on actin cytoskeleton (13). The actin cytoskeleton organization and dynamics are controlled by small-GTP-binding proteins, protein kinases and phosphatases, which regulate a multitude of actin cytoskeleton components, such as actin-polymerizing proteins (Arp2/3 complex, formins), actin-stabilizing proteins (α-actinin, filamins, tropomyosins), actin-associated proteins (HSP27, MLC2), and actin-severing proteins (gelsolin, cofilin). TGF-β promotes the disruption of cell-cell contacts either by altering the actin cytoskeleton (14) or by downregulating the expression of E-cadherin (15). Furthermore, TGF-β may positively or negatively control cell motility and matrix-degrading enzymes via tropomyosin-stabilized actin stress fibers (14,16). In carcinoma cells with low levels of tropomyosins, the upregulation of matrix-degrading enzymes, such as matrix metalloproteinases MMP-2 and MMP-9, participates in TGF-β induction of invasive behavior (7,17). MAP kinases have been involved in TGF-β regulation of the actin cytoskeleton and cell motility (3,14). Furthermore, oncogenic Ras-MAPK signaling interferes with the induction of EMT by TGF-β-Smad pathway (18), indicating that MAP kinase signaling may affect the outcome of TGF-β responses.

We have previously shown that highly invasive and metastatic murine mammary adenocarcinoma LM3 cells express TGF-β cytokines and receptors, and that they respond to TGF-β with enhanced invasion and secretion of matrix-degrading enzymes (19). The present study supports an autocrine role of TGF-β signaling in tumor progression, and explores mediators of the pro-oncogenic TGF-β activities in LM3 cells. Expression of kinase-inactive TGF-β receptors decreased both basal and TGF-β-induced invasion. Furthermore, the evaluation of signal-transduction mediators showed that p38MAPK and MEK contribute to TGF-β stimulation of cell motility and invasion. Experiments in syngeneic BALB/c mice showed that the expression of kinase-inactive TGF-β receptors decreased the tumorigenic potential of LM3 cells *in vivo*. Our study provides evidence for a role of MAP kinases in the pro-oncogenic activities of TGF-β in mammary tumor cells, including the regulation of the actin cytoskeleton, cell motility and invasion.

## Materials and methods

### Antibodies and other reagents

Human recombinant TGF-β1 protein was obtained from R&D Systems (#240-B). The following antibodies were used: Smad2 (34G6, #3107), phospho-Smad2 (#3101), phospho-ERK1/2 (#9101), phospho-p38MAPK (#9215), phospho-MLC2 (#3675), from Cell Signaling Technology; Smad4 for immunofluorescence (B-8, #sc-7966, Santa Cruz Biotechnology); Smad4/DPC4 for immunoblotting (BD Biosciences); β-actin (AC-40, #A4700), tropomyosin (TM311, #T-2780), anti-mouse/TRITC (#T-6653), anti-sheep/HRP (#A-3415), from Sigma; anti-rabbit/HRP (PI-1000) or anti-mouse/HRP (#PI-2000), from Vector Laboratories; BB-94, from British Biotech Pharmaceuticals. Alexa Fluor phalloidin was from Molecular Probes (#A-12379 or A-12380). Inhibitors for p38MAPK (SB202190, #559388), MEK1/2 (PD98059, #513000, or U0126, #662005), and Raf1 (5-iodo-3-[(3,5-dibromo-4-hydroxyphenyl)methylene]-2-indolinone, #553008) were from Calbiochem. The dual luciferase reporter assay system was from Promega (#E1910). The Arp2/3 complex kit, containing Arp3 antibody, was from Cytoskeleton, Inc. (#BK009).

### Cell lines and treatments

The mouse mammary hormone-independent adenocarcinoma LM3 cell line has been previously described (20). Non-tumorigenic murine mammary gland (NMuMG) cells were from ATCC (CRL-1636, ATCC). Mv1Lu cells were a gift from Dr Harold Moses. NMuMG cells and Mv1Lu cells were used as control for TGF-β response. LM3 and NMuMG cells were grown in medium supplemented with fetal bovine serum (5% FBS MEM or 10% FBS DMEM, respectively) with the addition of 80 μg/ml gentamycin. Mv1Lu cells were grown in 10% FBS DMEM supplemented with 3.7 g/l sodium bicarbonate. All cells were kept at 37°C in a humidified atmosphere with 5% CO_2_. Cells in exponential growth phase were treated with 2 ng/ml TGF-β1. For some assays, 1 ng/ml TGF-β1 was used. Kinase inhibitors were added to cells 1 h prior to TGF-β treatment, at the following doses: 10 μM SB202190; 5 μM PD98059; 5 μM U0126, and 5 μM c-Raf1 inhibitor.

### Retroviral infection of cells

Retroviral vectors used in the study are described in (21,22). Briefly, these vectors encode: EGFP in pBMN-IRES-EGFP (control); dominant-negative TβRI-K232R mutant and EGFP in pBMN-TβRI-K232R-IRES-EGFP; dominant-negative TβRII-K277R and EGFP in pGABE-TβRII-K277R. Amphotropic retroviruses were prepared as described in (21). Cells were infected with each retrovirus using polybrene (10 μg/ml, Sigma), and EGFP-positive cells were selected three consecutive times by FACS in order to obtain cell populations with similar levels of EGFP expression.

### Transcriptional analysis

Exponentially growing cells were transfected with 0.1 μg/ml of the following plasmids: pSBE-Lux, containing 12 repeats of Smad-binding sites (generously provided by J. M. Gauthier, Laboratoire Glaxo Wellcome, Les Ulis Cedex, France), or p3TP-Lux, containing 3 AP-1 sites and a fragment of the human PAI-1 promoter (23). Cells were co-transfected with 0.002 μg/ml pCMV-Renilla luciferase (Promega) using FuGENE6 (Roche Molecular Biochemicals), according to the manufacturer’s protocol. Cells were incubated in 0.5% FBS for 6 h prior to 1 ng/ml TGF-β1 treatment for 16 h. Luciferase activity in cell lysates was determined by the Dual Luciferase Reporter Assay system, according to the manufacturer’s protocol, using a Monolight 2010 luminometer (Analytical Luminiscence Laboratory). Firefly luciferase activity was normalized to Renilla activity, and expressed as luciferase relative units (LRU).

### RT-PCR analysis

Total RNA was prepared as previously described (24). RT-PCR was performed using One-Step RT-PCR system (Invitrogen). Amplification products were separated on 1% agarose gels and visualized with ethidium bromide. Primer sequences were mouse MMP-9 (NM_ 013599.2), forward CGTCGTGATCCCCACTTACT and reverse, AGGAAGACGAAGGGGAAGAC; α-tropomyosin (NM_024427.2): forward, GCTGGTGTCACTGCAAAAGA and reverse, CCTGAGCCTCCAGTGACTTC; mouse β-actin (NM_007393): forward, GCTGGTCGTCGACAACGGCTC and reverse, CAAACATGATCTGGGTCATCTTTTC.

### Western blot analysis

Cells were treated with TGF-β1 for different periods of time, and then lysed in buffer containing 20 mM Tris, pH 7.4, 137 mM NaCl, 1% NP-40, 10% glycerol, 20 mM NaF, 1 mM Na orthovanadate, 1 mM PMSF, 2 μg/ml aprotinin, and 2 μg/ml leupeptin. For signal transduction studies, cells were serum-starved for 4 h prior to treatment with TGF-β. Immunoblot analysis of protein extracts was performed as pre-viously described (19).

### Actin cytoskeleton study

Cells were grown on glass coverslips for 24 h prior to treatment with TGF-β1, and then fixed with 4% paraformaldehyde, permeabilized with 0.05% Triton X-100 in PBS for 15 min, and stained as described before (21). Actin filaments (F-actin) were visualized with Alexa Fluor phalloidin. Fluorescence images were captured using a Zeiss Axiophot upright microscope and a Nikon TE2000-E inverted microscope.

### Affinity purification of Arp2/3 complex activity

Activation of the Arp2/3 complex was examined using a pull-down assay kit from Cytoskeleton, Inc., following the manufacturer’s protocol. Briefly, cells were incubated for 4 h in serum-free medium prior to treatment with TGF-β1, and then lysed. Total proteins were incubated with either GST-VCA beads, in order to precipitate active Arp2/3 complex, or with GST beads alone as a control. Pellets containing the Arp2/3 complex were analyzed for Arp2/3 activity by immunoblotting with anti-Arp3 antibody. Supernatants were also examined, as a control.

### Zymography for metalloproteinase (MMP) activity

MMP-9 activity was measured by quantitative gelatin zymography of conditioned media (CM) from cells treated with or without TGF-β1, as previously described (19). Gelatinolytic bands were analyzed by the GS-700 densitometer and the Molecular Analyst™ software (Bio-Rad), and OD values were used as a measurement of total cellular protein content.

### Cell migration

Cell migration was studied in a wound healing assay, as previously described (19). Briefly, cells were cultured until confluency and wounds of ~400 μm width were made on the monolayers with a plastic tip. Then, cells were incubated with TGF-β1 for another 16 h. Photographs of the same area were taken at ×400 magnification to determine wound coverage due to cellular motility. Images were obtained and evaluated by densitometry, using Image-Pro Plus 5.1 software.

### Invasion assay

Cell invasion assays were performed using Matrigel-coated Transwell chambers (8 μm filter pore, Corning), as previously described (19). Cells were seeded onto Transwell chambers and incubated with or without TGF-β1 for 16 h (cytokine added in the plate well). Cells on the bottom surface of the filter (those who had traversed the filter) were stained with Hoechst 33258 (10 μg/ml, Sigma), and counted under fluorescence microscope at ×600 magnification.

### Tumor growth and metastatic ability

LM3 stably cells expressing TβRI-K232R, TβRII-K277R, as well as control cells expressing EGFP, were harvested at the exponential growth phase with trypsin/EDTA, washed and resuspended in MEM. Cells (2×10^5^) in 0.2 ml MEM were inoculated subcutaneously into the flank of syngeneic BALB/c mice (10 female mice per group). Tumor latency was defined as the time between inoculation and detection of tumors by palpation. Tumor size was measured with a caliper, in orthogonal directions, every 3 days. Animals were euthanized on day 40 of tumor onset, time at which spontaneous superficial lung metastases are detected in this tumor model. The condition of every major organ was observed, the lungs were removed, fixed with Bouin’s solution, and examined under a magnifier to record the number and size of metastatic foci. Both tumors and lungs were analyzed *ex vivo* under fluorescence microscope to determine the presence of EGFP-positive cells.

### Statistical analysis

In general, all experiments were performed at least three times, and the mean value of triplicates in each comparable group was analyzed using the Student’s t-test or the ANOVA-Scheffé’s test. Differences in metastatic ability between the groups were investigated using the non-parametric Mann-Whitney U test. Results were considered of biological significance when p<0.05.

## Results

### Expression and activation of Smads and MAPK pathways

**Figure 1:**
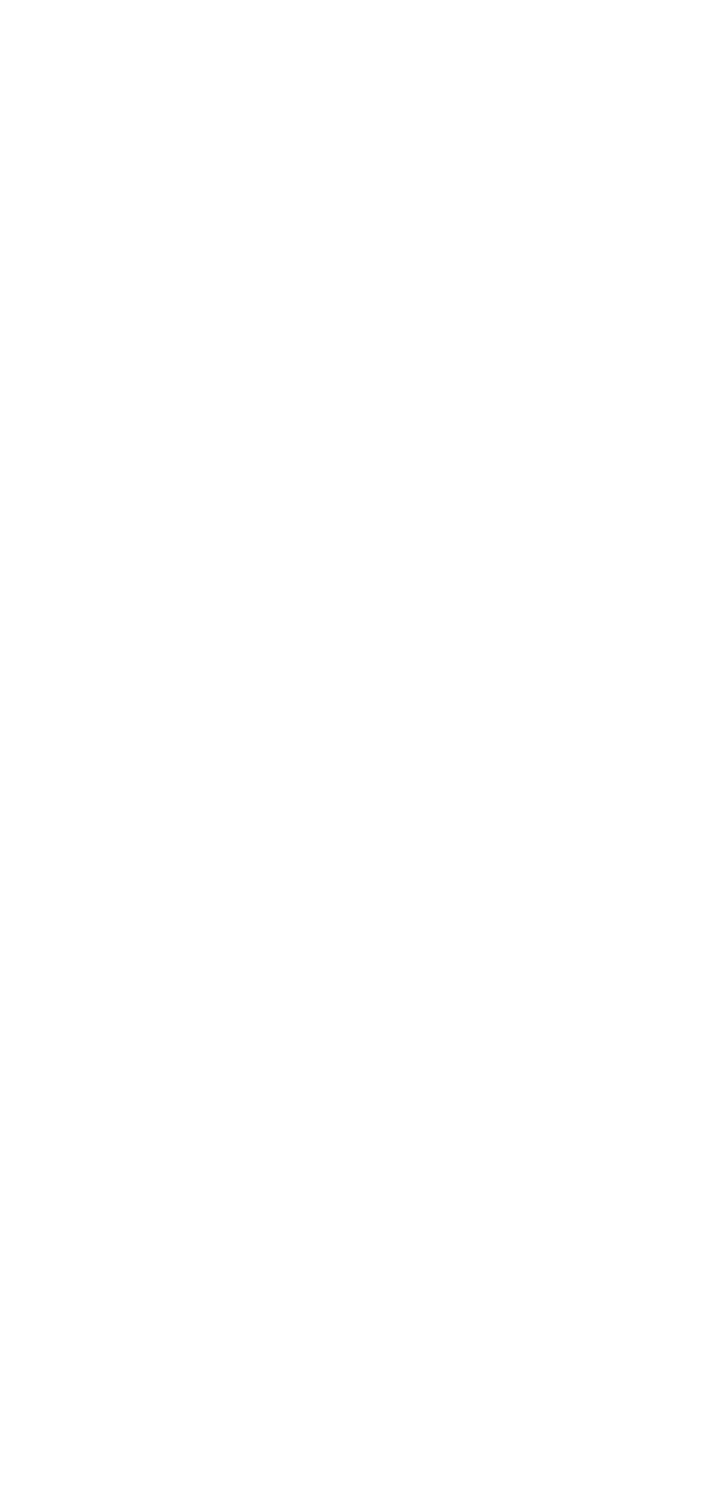
The TGF-β pathway and other intracellular signaling pathways in LM3 cells. (A) Immunoblot analysis of phosphorylated Smad2, p38MAPK and ERK1/2, as well as the levels of total Smad2 and Smad4 in LM3 cells. Actin was used as loading control. (B) Smad4 nuclear localization by immunofluorescence in LM3 cells treated with or without 2 ng/ml TGF-β1 (magnification, ×1000). Mv1Lu cells were used as a control of the TGF-β response. White arrows show Smad4 nuclear localization. (C) Smad-dependent transcriptional activity in LM3 cells in response to 1 ng/ml TGF-β1 for 16 h. Prior to treatment, cells were co-transfected with luciferase reporters SBE-Lux and 3TP-lux (see Materials and methods). NMuMG cells were employed as a control (inset). Experiments were performed in triplicates and repeated at least twice. Data represent the mean ± SD of luciferase relative units (LRU) from triplicates. (D) PAI-1 mRNA expression in LM3 cells treated with or without 2 ng/ml TGF-β1 for 8 or 24 h, by RT-PCR. NMuMG cells were used as control of TGF-β response. β-actin (Actb) was employed as a control.

The regulation of MAP kinase and Smad pathways by TGF-β in the mammary adenocarcinoma LM3 cells was evaluated by immunoblotting and immunofluorescence. Immunoblot analysis revealed that TGF-β treatment increased phosphorylation of Smad2 between 30 min and 4 h, while total levels of Smad2 and Smad4 were not changed for up to 24 h treatment (Fig. 1A). In Fig. 1B, immunofluorescence showed nuclear translocation of Smad4 at 30 min of TGF-β treatment, indicating activation of the Smad complex in response to TGF-β. Concomitantly, TGF-β induced the phosphorylation of p38MAPK and ERK1/2 (Fig. 1A).

TGF-β transcriptional responses were evaluated using a luciferase reporter containing 12 repeats of Smad-binding sites (SBE-Lux) and a reporter containing a fragment of the PAI promoter and 3 repeats of AP1 sites (3TP-Lux) in LM3 cells. NMuMG cells, which display a strong regulation by both reporters, were used as the control (14). As shown in Fig. 1C, TGF-β1 significantly increased the activity of both reporters in LM3 cells. In addition, RT-PCR analysis showed that TGF-β treatment upregulated endogenous PAI-1 mRNA levels (Fig. 1D). Together, these findings demonstrate that LM3 cells respond to TGF-β with activation of the Smad and MAPK signaling pathways. As evidenced by the modulation of downstream targets, such as PAI-1, these pathways are functional.

### TGF-β signaling enhances LM3 cells invasive ability

Our previous studies have shown that the LM3 cell line expresses TGF-β cytokines and TGF-β receptors, and is able to respond to TGF-β with enhanced invasion *in vitro*(19). Here, LM3 cells were transduced with retroviral vectors to express dominant negative (kinase-inactive) forms of TGF-β type I and type II receptors (TβRI-K232R and TβRII-K277R, respectively) by retroviral infection using bicistronic EGFP-encoding vectors (21). These kinase-inactive receptors exert dominant-negative effects on TGF-β signaling (7,21). An EGFP-encoding vector was used as a control.

**Figure 2:**
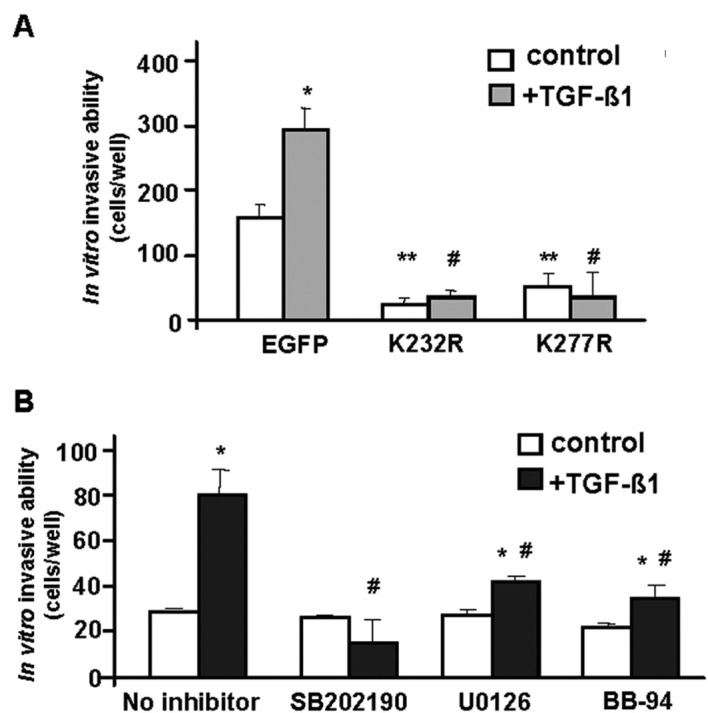
Autocrine TGF-β signaling and MAPK pathways promote invasiveness in LM3 murine mammary adenocarcinoma cells. (A) LM3 cells expressing dominant-negative TGF-β receptors type I, TβRI-K232R, or type II, TβRII-K277R, as well as EGFP-expressing control cells were grown in Matrigel-coated Transwell chambers. Cells were treated with or without 2 ng/ml TGF-β1 (see Materials and methods). Cells that had invaded the Matrigel and had migrated to the bottom side of the Transwell filter after 16 h of treatment were counted. Experiments were performed in triplicates and repeated at least twice. Data represent the mean ± SD of triplicates. (^*^p<0.05 vs. the untreated cells; ^**^p<0.05 vs. the LM3/EGFP untreated cells; ^#^p<0.05 vs. the TGF-β-treated LM3/EGFP cells). (B) Matrigel invasion assay was performed with LM3 cells in the presence of the p38MAPK inhibitor SB202190 (10 μM), the MEK inhibitor U0126 (5 μM) or the metalloproteinase inhibitor BB-94 (5 μM). (^*^p<0.05 vs. the untreated cells; ^#^p<0.05 vs. the TGF-β-treated cells).

The invasive ability of cells expressing TGF-β kinase-inactive receptors was significantly impaired in the absence of exogenous ligand (compare to EGFP alone vector), indicating that autocrine TGF-β signaling could contribute to the basal invasive properties of LM3 cells (Fig. 2A). In addition, while TGF-β1 stimulated the number of LM3-EGFP cells invading the Matrigel-coated chambers by ~2-fold (Fig. 2A), kinase-inactive receptors blocked TGF-β-induced invasion in LM3 cells.

This experiment was also performed in the presence of pharmacological inhibitors of cell signaling pathways. Inhibition of p38MAPK with SB202190, and of MEK-ERK signaling with U0126, blocked the TGF-β1-induced invasive ability in LM3 cells (Fig. 2B). Similar results were obtained with a metalloproteinase inhibitor, BB-94 (Fig. 2B). These findings suggest that the p38MAPK as well as the MEK-ERK signaling pathways are required for TGF-β regulation of invasiveness in mammary tumor cells. Moreover, the results indicate a putative synergistic role with metalloproteinases.

### TGF-β modulation of matrix metalloproteinase 9/gelatinase-B (MMP-9)

The invasive ability of cells depends on cell motility and on the activity of matrix-degrading enzymes. Our previous studies have demonstrated that LM3 cells express MMP-9 and that TGF-β markedly enhances the secreted MMP-9 activity (19). Here, we explored the mechanism underlying this TGF-β effect.

**Figure 3:**
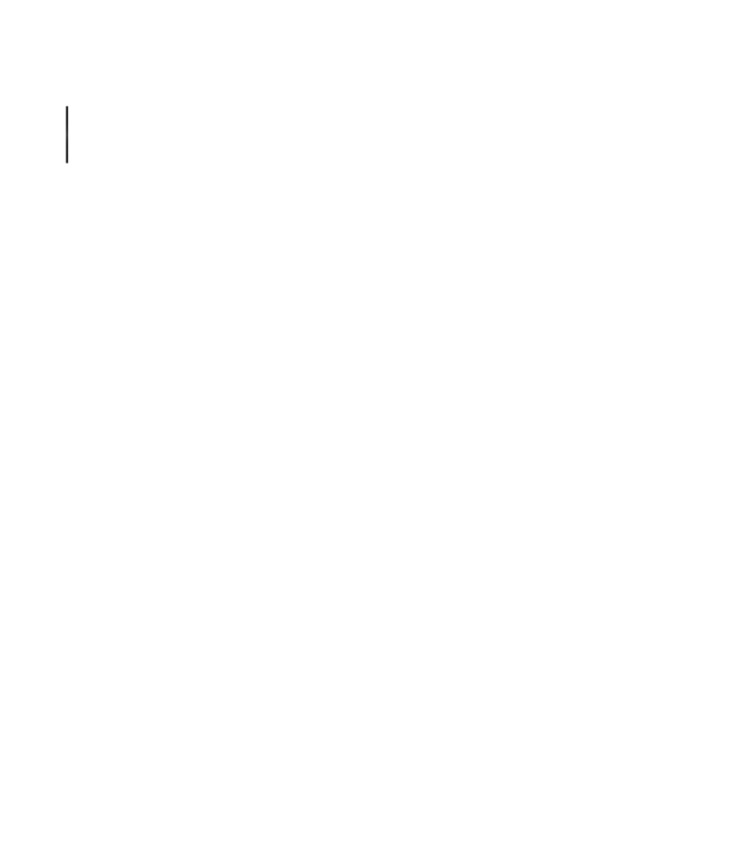
TGF-β upregulates MMP-9 secretion in LM3 tumor cells. (A) MMP-9 mRNA expression in LM3 cells treated with 2 ng/ml TGF-β1, by RT-PCR. β-actin (Actb) was used as loading control. (B) MMP-9 activity in LM3 cells treated with or without 2 ng/ml TGF-β1, by gelatin zymography of 24 h conditioned medium. Control EGFP-expressing LM3 cells, as well as LM3 cells expressing dominant-negative receptors TβRI-K232R or TβRII-K277R, were also examined. (C) Gelatinase activity in LM3 cells in response to TGF-β1, in the presence of the p38MAPK inhibitor SB202190 (10 μM) or the MEK inhibitor PD98059 (5 μM). In both (B) and (C), data are expressed as the mean ± SD of triplicates, and are representative of three independent experiments. (^*^p<0.05 vs. the untreated cells; ^#^p<0.05 vs. the TGF-β-treated cells).

RT-PCR analysis showed that TGF-β1 upregulated MMP-9 mRNA level within 8 h of treatment (Fig. 3A). Gelatin zymography assays revealed that dominant-negative TGF-β receptors blocked the stimulation of secreted MMP-9 by TGF-β in LM3 and LM3-EGFP (control) cells (Fig. 3B). In order to identify signaling pathways involved in MMP-9 induction by TGF-β, the same experiment was performed in the presence of kinase inhibitors. Surprisingly, we observed that both the p38MAPK inhibitor SB202190 and the MEK inhibitor PD098059 enhanced the TGF-β-induced MMP-9 activity (Fig. 3C), suggesting the antagonic effects between the p38MAPK and MEK pathways and the TGF-β pathways on MMP-9 secretion by LM3 cells.

### Modulation of cell motility by TGF-β requires MAP kinases

**Figure 4:**
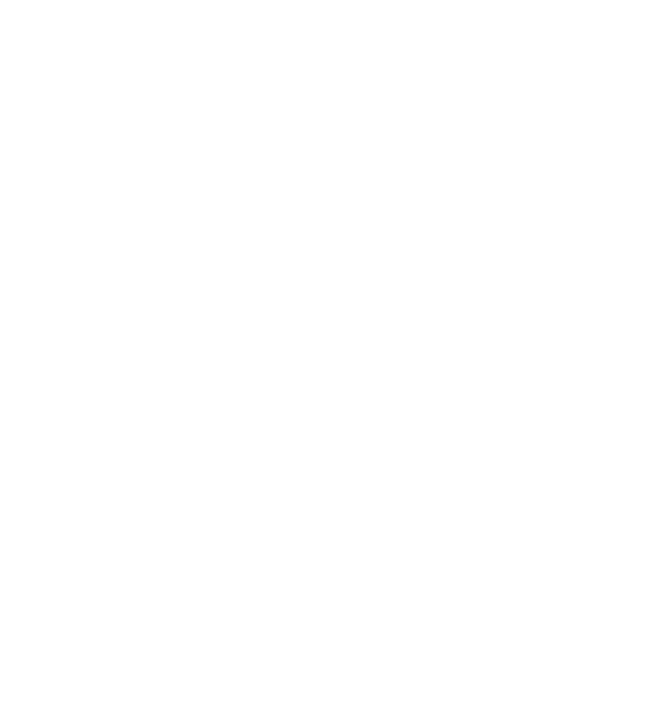
TGF-β regulates motility in LM3 cells. (A) LM3 cells treated with 2 ng/ml TGF-β1 for 16 h, by the wound-healing assay. Cells were also incubated with the kinase inhibitors SB202190 (10 μM) or U0126 (5 μM). Microphotographs of the same wound area were taken at the time of wounding and 16 h thereafter. Experiments were performed in triplicates and repeated at least twice. Representative phase-contrast images at ×40 magnification are shown. (B) Evaluation of cell migration. Data are expressed in arbitrary units (AU), and represent the mean ± SD of triplicates. (^*^p<0.05 vs. untreated cells, ^#^p<0.05 vs. TGF-β-treated LM3 cells).

The effect of TGF-β on cell motility was evaluated by wound assay. Incubation of LM3 cells with 2 ng/ml TGF-β1 significantly accelerated the healing of wounds in cell monolayers, indicating that TGF-β1 enhanced cell motility (Fig. 4A). To assess the role of MAPK signaling in TGF-β-induced motility in these cells, wound assays were performed in the presence of p38MAPK and MEK inhibitors. The inhibition of either kinase significantly abrogated TGF-β induction of wound healing (Fig. 4B), suggesting that both p38MAPK and MEK-ERK pathways are involved in the regulation of LM3 cell motility by TGF-β1.

### Effect of TGF-β on LM3 cytoskeleton

The regulation of cell motility by TGF-β in normal and tumor cells has been linked to EMT, which involves the disruption of cell-cell junctions as well as actin remodeling (15) and, in some cases, it also involves actin-stabilizing proteins such as high molecular-weight (HMW) tropomyosins. Therefore, in order to investigate the mechanism of TGF-β-mediated cell migration in LM3 cells, we assessed the regulation of actin cytoskeleton and HMW-tropomyosins.

**Figure 5:**
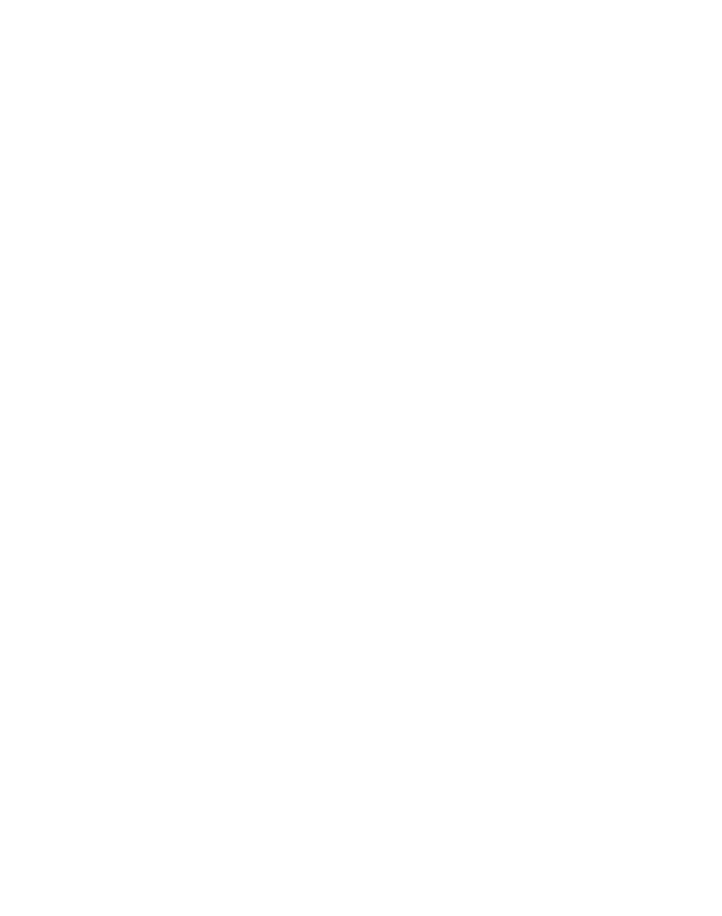
TGF-β regulates the actin cytoskeleton and Arp2/3 complex activity. (A) Phalloidin staining of actin filaments in cells treated with 2 ng/ml TGF-β1 for 24 h, either alone or in the presence of the p38MAPK inhibitor SB202190 (10 μM), the MEK inhibitor U0126 (5 μM) or the Raf1 inhibitor (5 μM). Arrows indicate the disruption of actin adhesion belts in LM3 cells treated with TGF-β. Note extensive actin stress fibers in TGF-β-treated cells in the presence of MEK or Raf inhibitor. (B) Tropomyosin (TPM) immunoblot in LM3 cells in response to 2 ng/ml TGF-β1. NMuMG cells were used as a control. (C) Phosphorylated MLC2 immunoblot in response to 2 ng/ml TGF-β1. (D) Arp2/3 complex activity in LM3 cells treated with or without 2 ng/ml TGF-β1, by an affinity pull-down assay. Precipitates (P) or supernatants (SN) were analyzed with Arp3 antibodies. (E) Arp2/3 complex activity in response to TGF-β, in the presence of the kinase inhibitors SB202190 (10 μM) or U0126 (5 μM).

Immunofluorescence microscopy showed the organization of actin filaments in adhesion belt-like structures in control LM3 cells, typical of epithelial cells (Fig. 5A). Treatment with TGF-β1 for 24 h disrupted these structures and increased linear actin filaments (Fig. 5A). Co-incubation with a p38MAPK inhibitor blocked actin remodeling in response to TGF-β, whereas co-incubation with a MEK inhibitor (U0126) markedly enhanced the TGF-β-induced increase of actin stress fibers (Fig. 5A). Similar results were obtained with a Raf1 inhibitor, corroborating that the inhibition of another molecule of the Ras/MAPK/ERK pathway positively stimulates the formation of stress fibers in the presence of TGF-β.

On the other hand, immunoblotting showed that TGF-β did not modulate the expression of HMW-tropomyosins (TPMs), contrasting our findings in NMuMG cells, employed as a positive control (Fig. 5B). Moreover, the mRNA levels of TPM-α and TPM-β genes were not regulated by TGF-β in LM3 cells (data not shown).

We further assessed TGF-β modulation of the actin cytoskeleton by analyzing the activity of myosin II regulatory light chain (MLC2), which regulates actomyosin contractility and cell migration (13). The immunoblots in Fig. 5C show the induction of MLC2 phosphorylation and activation within 30 min of TGF-β treatment, which persisted for 24 h, indicating that TGF-β increased actomyosin contractility in LM3 cells.

Arp2/3 protein complex mediates *de novo* actin filament nucleation during polymerization of branched actin structures (13). We thus analyzed whether the function of this complex is affected by TGF-β. The Arp2/3 activity complex was assessed by a pull-down assay using GST-VCA fusion proteins in which the C-terminal VCA domain of WASP was linked to GST. A conserved VCA domain of WASP contains a verprolin homology segment (V), a cofilin homology segment (C) and an acidic region (A). This domain interacts and activates the Arp2/3 complex (13). We found that TGF-β increased the association of the Arp2/3 complex with the VCA domain within 10–30 min of treatment in LM3 cells (Fig. 5D). This response was not affected by p38MAPK or MEK kinase inhibitors (Fig. 5E).

These findings suggest that the mechanism of TGF-β stimulation of cell motility in LM3 cells may involve actin remodeling, actomyosin contractility, and Arp2/3 complex activity.

### TGF-β signaling in tumor development and progression

**Figure 6:**
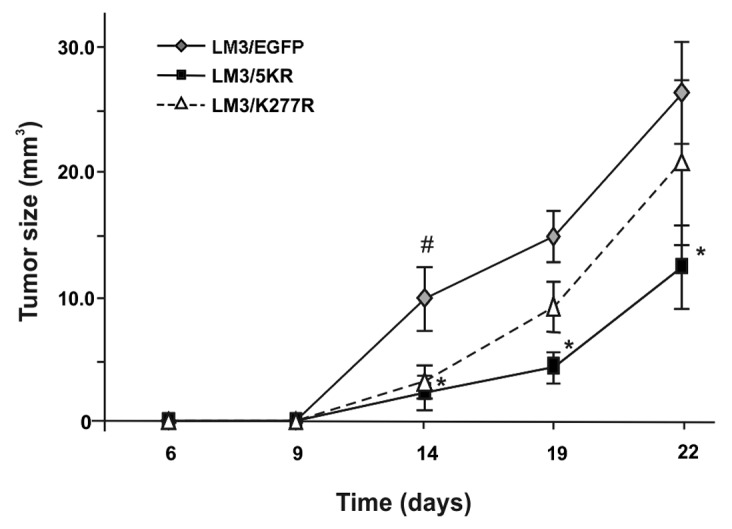
Effect of dominant-negative TGF-β receptors on tumor growth. *In vivo* tumor growth of LM3 cells expressing TβRI-K232R (LM3/K232R) or TβRII-K277R (LM3/K277R), or EGFP-control vector (LM3/EGFP). Cells were inoculated s.c. into syngeneic BALB/c mice, and tumor size was measured every 3 days. Data for the first 25 days after inoculation are expressed as the mean ± SD (10 animals per group), and are representative of two independent experiments. (^*^p<0.05 vs. LM3/EGFP cells).

To examine the role of TGF-β signaling pathway in tumor growth and spontaneous metastasis, we employed syngeneic BALB/c mice. LM3 cells expressing TβRI-K232R (LM3/K232R), TβRII-K277R (LM3/K277R) or EGFP-control (LM3/EGFP) were injected subcutaneously into the flank of syngeneic BALB/c mice. All three groups of mice developed palpable tumors within a week of tumor cell inoculation, and showed comparable tumor incidence (90–100%) as well as latency period. However, we observed that tumor growth during the first ~25 days was significantly reduced in both dominant-negative TGF-β receptor groups (Fig. 6). We continued our observations on tumor growth until day 40, at which time the animals were sacrificed. The dominant negative TβR bearing-LM3 tumors were smaller in size than the control tumors (LM3/EGFP), although the values did not reach statistical significance (data not shown). In addition, no difference was observed in the number of lung foci at the latest time point analyzed (40 days after tumor cell inoculation). The median number of metastases were 5 (range, 2–32) in the control group; 5 (0–11) in the K232R group, and 9 (0–56) in the K277R group.

## Discussion

In our model of murine mammary LM3 adenocarcinoma cells, TGF-β triggered the activation of Smad and non-Smad signaling pathways, with the upregulation of downstream targets, such as PAI-1, confirming the functionality of the TGF-β/TβR system in these cells. We demonstrate that TGF-β enhances LM3 cell motility, inducing actin remodeling, actomyosin contractility, and the activation of the Arp2/3 complex. Our results show that the non-Smad downstream effectors p38MAPK and MEK/ERK regulate actin remodeling and cell motility, but do not contribute to the regulation of the Arp2/3 complex. In addition, TGF-β also induced the matrix-degrading ability of LM3 cells mediated by MMP-9 secretion. Expression of kinase-inactive dominant-negative TGF-β receptors markedly reduced the invasive potential of LM3 cells, indicating that an autocrine TGF-β signaling loop may contribute to the invasive phenotype. Moreover, we show that TGF-β signaling may be a determinant of initial tumor growth *in vivo*.

In order to invade and spread to distant organs, carcinoma cells must lose polarity, cell-cell contacts, and acquire fibroblastic-like properties. In this process of epithelial-mesenchymal transition (EMT), cells become highly motile and invasive, which allows survival in an anchorage-independent environment and provides them with stem cell-like properties. The activity of proteases such as MMPs, which leads to the degradation of extracellular matrix proteins, may render tumor cells with a migratory and invasive advantage. Our results show that TGF-β presents pro-invasive and pro-migratory effects on LM3 cells. Similar results were observed in other murine and human mammary carcinoma cell lines (8,25–27).

It has been established that even though Smad signaling is required for the majority of TGF-β-mediated signals, not all responses to TGF-β are solely dependent on the Smad complex. In fact, the TGF-β response implies alternative signaling modules acting in parallel with Smads. As an example, it was demonstrated that TGF-β signaling is engaged in RhoA-ROCK signaling, required for the regulation of cell shape and movement (28,29). In addition, TβRI activates ERK-MAPK signaling through direct phosphorylation of Shc, and TβRII can signal independently of TβRI by directly phosphorylating Par6, an EMT-associated biomarker that enhances proliferation, migration and invasiveness in cells *in vitro*(30). Thus, the signaling pathways triggered by TGF-β/TβR signaling are pliable and diverse. Here, we demonstrate that intracellular signaling by p38MAPK and MEK is involved in both the pro-invasive and the pro-migratory activities of TGF-β in LM3 cells. Dumont *et al* have also found that these two signaling pathways mediate the pro-invasive effect of TGF-β on the human ER-negative MDA-MB-231 cell line (26).

On the other hand, we found that p38MAPK and MEK have distinct effects on TGF-β-induced disruption of the epithelial actin cytoskeleton and cell-cell junctions, and on the formation of actin fibers, which are key aspects of EMT (15). Our findings indicate that p38MAPK is required for the disruption of epithelial organization of actin filaments and cell-cell junctions. This result is in agreement with previous studies showing that p38MAPK is required for the TGF-β-induced EMT process, and for actin remodeling on the non-tumorigenic NMuMG cells (21,31). Surprisingly, we found that blocking MEK1/2 significantly increased the formation of stress fibers, similarly to a Raf1 inhibitor, a MEK upstream molecule. These results indicate that Raf-MEK-ERK signaling suppresses TGF-β-induced actin stress fibers formation in LM3 cells. Other authors, employing a similar approach, demonstrated that TGF-β1 induces the activation of the ERK signaling pathway in NMuMG cells, which is required for TGF-β1-mediated EMT *in vitro*(32). Therefore, it appears that the signaling pathways activated by the TβR are highly dependent on cell properties.

Stimulation of cell motility by TGF-β is a complex process involving several factors. In our model, in addition to the disruption of the actin filaments architecture, TGF-β increased phosphorylation of the regulatory subunit of the actomyosin contractility complex MLC2, thus enhancing actomyosin contractility, and ultimately contributing to cell motility and cell-matrix adhesion (33,34). MLC2 phosphorylation is controlled by RhoA-ROCK signaling, Pak1, MLC kinase and phosphatase (34) and TGF-β may be regulating MLC2 phosphorylation via RhoA-ROCK and Pak1 signaling (29,35,36). Moreover, in our study we made the novel observation that TGF-β regulates the Arp2/3 complex, which is also related to cell motility and invasion (13,34). The Arp2/3 complex mediates actin nucleation enabling *de novo* polymerization of actin filaments (13,37). We found that TGF-β rapidly activates Arp2/3 complex in LM3 cells, and that the p38MAPK or MAPK-ERK pathways are not involved in this TGF-β effect. To the best of our knowledge, this is the first report of Arp2/3 complex activation by TGF-β. It is known that Arp2/3 complex activation may involve other proteins, such as WASP (Wiskott–Aldrich syndrome protein)/WAVE3 proteins, which are activated by Rac and CDC42 GTPases (13). On the other hand, TGF-β can rapidly activate the Rho-family GTPases, Rac1, CDC42, and RhoA, in normal and tumor cells, although the mechanism is still unknown (21,35,36,38). WAVE3 is frequently upregulated in mammary carcinomas and it may contribute to the regulation of p38MAPK (39). Thus, the activation of the Arp2/3 complex by TGF-β may involve Rac1/CDC42 and WASP/WAVE3 proteins. Further studies may help elucidate the mechanisms of this TGF-β response, but our observations suggest that TGF-β may have a more profound effect on the actin machinery than previously thought.

The formation of actin fibers also requires Smad- and p38MAPK-dependent expression of HMW-tropomyosins (14). In the non-tumorigenic NMuMG cells, TGF-β upregulates HMW-tropomyosins, and it inhibits cell invasion (18), whereas in LM3 tumor cells, TGF-β does not modulate HMW-tropomyosins but stimulates cell invasion. It appears that the difference may be linked to MEK-ERK signaling. Active Ras-MEK-ERK signaling inhibits Smad activity (40,41), and overexpression of oncogenic RasV12 in NMuMG cells represses the TGF-β-Smad-mediated induction of HMW-tropomyosins and actin fibers but enhances cell motility and invasion (18). Furthermore, siRNA-mediated suppression of HMW-tropomyosins inhibits formation of actin stress fibers during the EMT process (14), whereas expression of tropomyosin enhances actin fibers and inhibits Ras-mediated cell transformation (18). Similar observations have been reported for breast cancer MDA-MB-231 and murine carcinoma 4T1 cell lines, which express high levels of active Ras-MEK-ERK signaling (8,14,25,27). Smad4 knock-out accelerates the development of pancreatic ductal carcinomas and metastases in the context of K-RasG12D transgenic mice (42,43). Together, these findings suggest that TGF-β pro-oncogenic activities in tumor cells are associated with reduced Smad-dependent responses and elevated levels of MEK-ERK signaling.

We found that while TGF-β signaling enhances cell invasion and secretion of matrix metalloproteinase-9/gelatinase-B (MMP-9) in LM3 cells, the MMP inhibitor BB-94 decreases LM3 invasiveness. Solid evidence implicates MMPs in tumor invasion and metastasis, and the link between TGF-β and MMP-9 has been extensively studied (44,45). Interestingly, MMP-9 serves as both a downstream target of TGF-β as well as an activator of latent TGF-β. In another breast carcinoma model, MMP-9 regulation by TGF-β did not require p38MAPK (7,46). However, by using kinase inhibitors, we observed an antagonic effect between the p38MAPK and MEK pathways and TGF-β signaling on MMP-9 secretion in LM3 cells. More studies are currently in progress to elucidate this mechanism.

Studies in both animal and human tumors have suggested an active role for TGF-β during *in vivo* tumor dissemination. Moreover, some breast cancer metastases have higher TGF-β immunostaining than primary tumors (11). Our *in vivo* studies further support the important role of TGF-β in tumorigenesis, since the expression of dominant-negative TGF-β receptors, which disrupts TGF-β signaling, significantly delayed initial LM3 tumor growth in syngeneic mice. Even though this effect was diluted with tumor evolution, our results allow us to speculate the TGF-β implication in tumor development. Thus, in our mammary cancer model, LM3 cells seem to be dependent on a functional TGF-β signaling, together with p38MAPK and MEK, in order to acquire migratory and invasive abilities, which allows tumor growth *in vivo*. However, once the tumor is established and reaches log-phase growth, further tumor progression appears to become independent of TGF-β. The determinant signals during later steps of tumor growth as well as during tumor progression, remain to be unraveled.

In summary, our studies demonstrate the important role of TGF-β signaling, together with other intracellular pathways, in the invasive and migratory properties of LM3 mammary adenocarcinoma cells. TGF-β pro-tumorigenic activities were apparent through the regulation of the actin cytoskeleton, an increase in migratory and invasive abilities, and through the induction of tumor growth *in vivo*. Since the LM3 cell line is derived from a spontaneous mammary adenocarcinoma in BALB/c mice, it represents a useful and novel model for investigating the pro-oncogenic activities of cytokines.
